# Supplementary figures and images for: Noncanonical mechanism of Nrf2 activation by diacylglycerol polyethylene glycol adducts in normal human epidermal keratinocytes
Source: PLoS One. 2023 Oct 11;18(10):e0291905. doi: 10.1371/journal.pone.0291905 (PMC10566712; doi:10.1371/journal.pone.0291905)

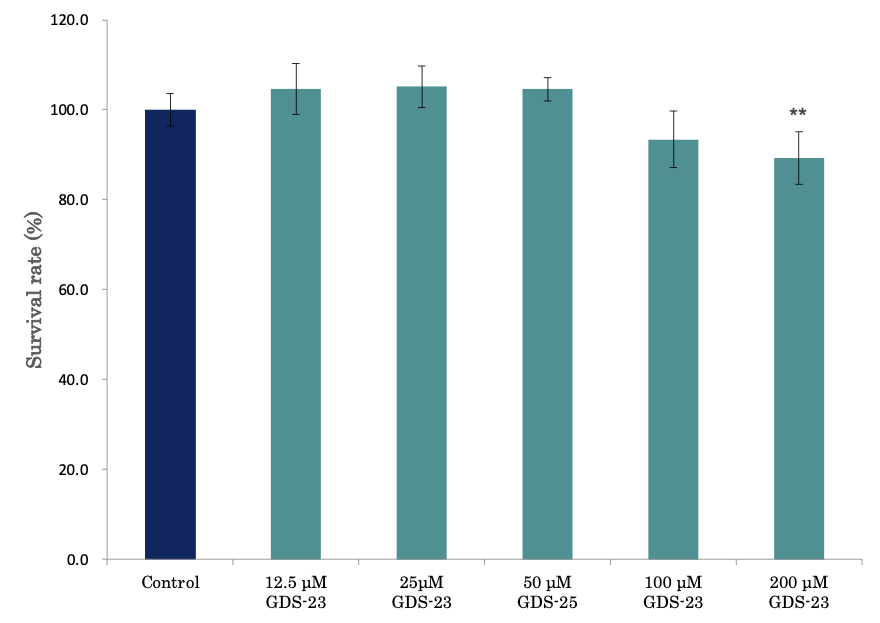

Supplement: S1 Fig — NHEKs were treated with GDS-23 at concentrations ranging from 12.5 to 200 μM for 24 h, and cytotoxicity was evaluated using a neutral red assay. Results are expressed as a percentage survival compared to the control cells and as mean ± S.D. (n = 6). Significance; **p < 0.01, ***p < 0.001 vs. control (Dunnett’s test). GDS-23, glyceryl polyethylene glycol-23 distearate; NHEK, normal human epidermal keratinocyte. (TIF) [file pone.0291905.s001.tif]

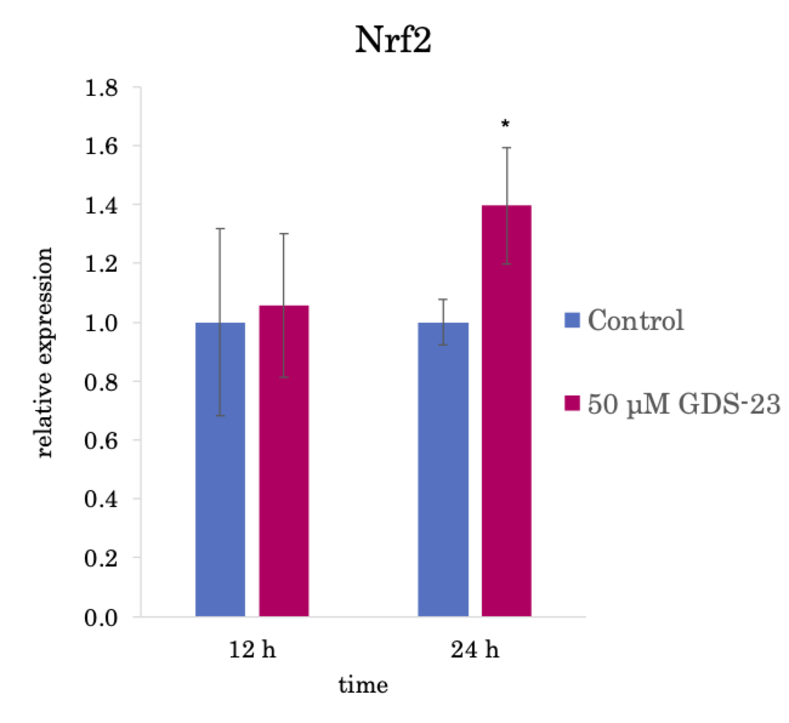

Supplement: S2 Fig — NHEKs were treated with 50 μM GDS-23 for 12 and 24 h. Control cells were treated with vehicle. Nrf2 mRNA levels were analyzed using qPCR. Results are expressed as mean ± S.D. (n = 4). Significance; *p < 0.05 vs. control cells (Student’s t-test). GDS-23, glyceryl polyethylene glycol-23 distearate; NHEK, normal human epidermal keratinocyte; Nrf2, nuclear factor E2-related factor-2. (TIF) [file pone.0291905.s002.tif]

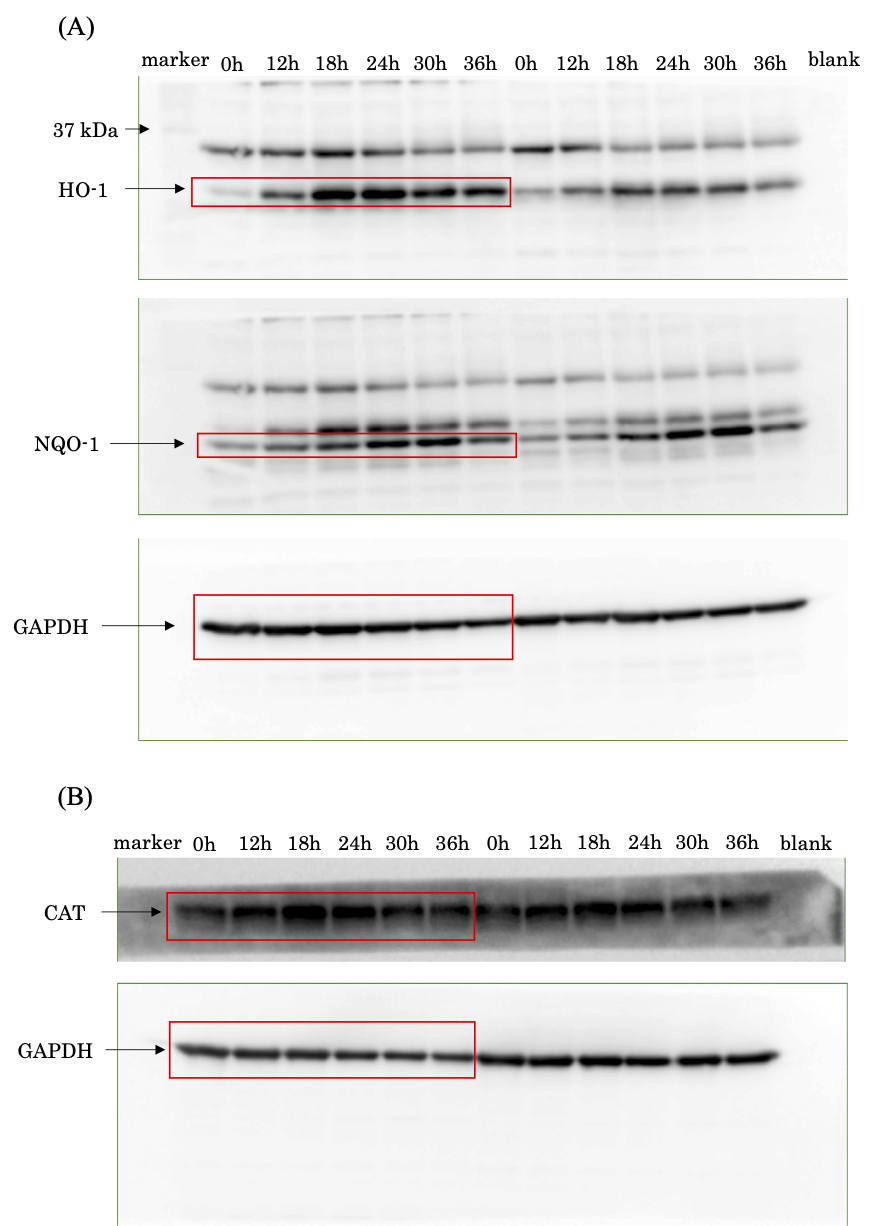

Supplement: S3 Fig — (A) To simultaneously or sequentially detect antigens using multiple antibodies, the membrane was cut below the 50 kDa marker. This was done to specifically focus on the detection of GAPDH (36 kDa), HO-1 (32 kDa), and NQO1 (31 kDa). The detection was performed first for HO-1, followed by NQO1, and finally GAPDH. After each detection, the antibodies were stripped from the membrane and the process was repeated for the next protein of interest. (B) To simultaneously detect antigens using multiple antibodies, the membrane was cut at two positions: below the 50 kDa marker and below the 75 kDa marker. This was done to specifically focus on the detection of GAPDH (36 kDa) and CAT (64 kDa). Red squares indicate the band position used in Fig 3A and 3B. (TIF) [file pone.0291905.s003.tif]

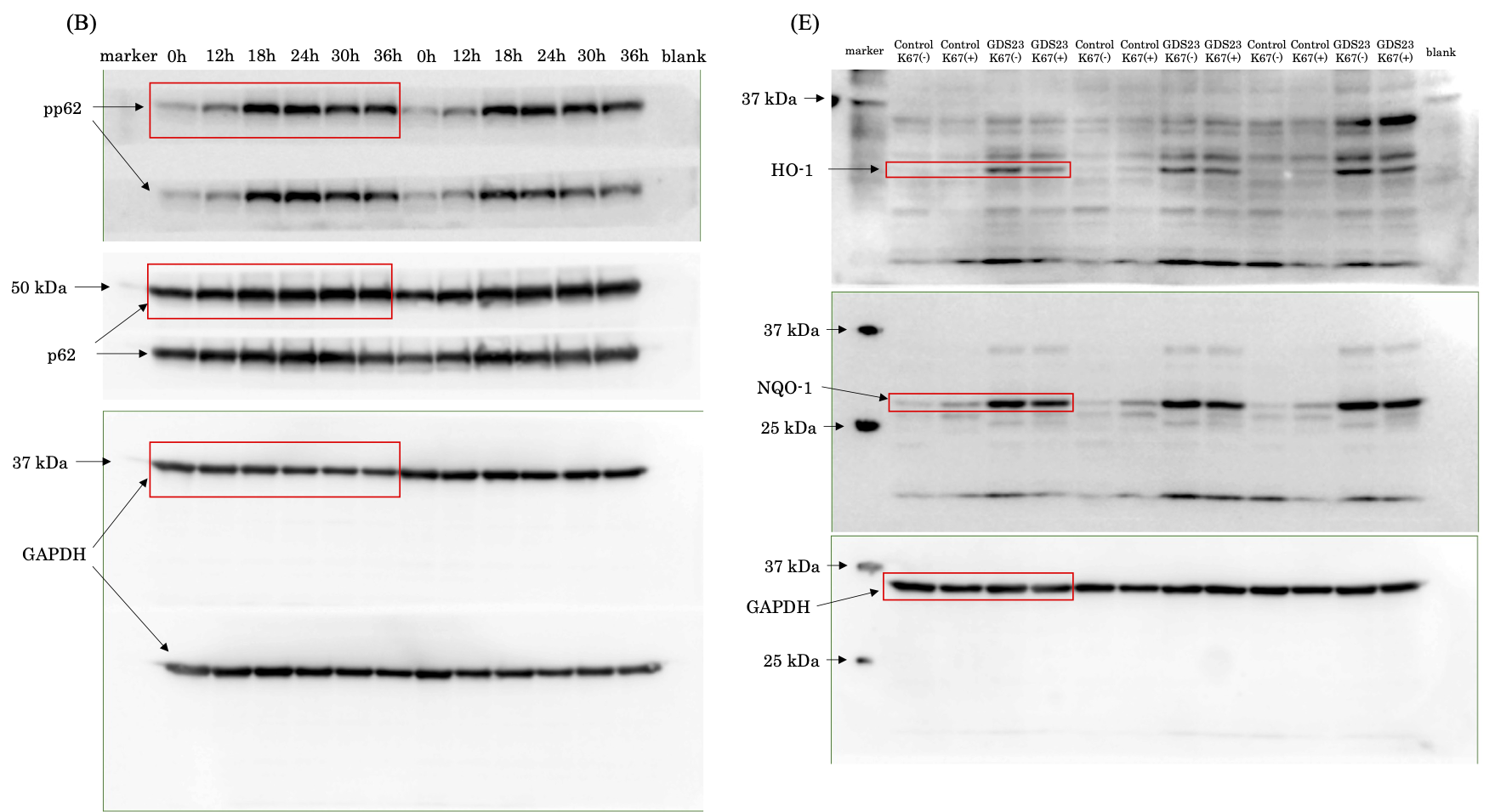

Supplement: S4 Fig — (B) To simultaneously or sequentially detect antigens using multiple antibodies, the membrane was cut at two line positions: below the 50 kDa marker and below the 75 kDa marker. This was done to specifically focus on the detection of GAPDH (36 kDa), p62 (62 kDa), and phosphorylated p62 (62 kDa). The detection was performed first for p62 and then for phosphorylated p62. After each detection, the antibodies were stripped from the membrane and the process was repeated for the next protein of interest. (E) To simultaneously or sequentially detect antigens using multiple antibodies, the membrane was cut below the 50 kDa marker. This was done to specifically focus on the detection of GAPDH (36 kDa), HO-1 (32 kDa), and NQO1 (31 kDa). The detection was performed first for HO-1, followed by NQO1, and finally GAPDH. After each detection, the antibodies were stripped from the membrane and the process was repeated for the next protein of interest. Red squares indicate the band position used in Fig 5B and 5E. (TIF) [file pone.0291905.s004.tif]

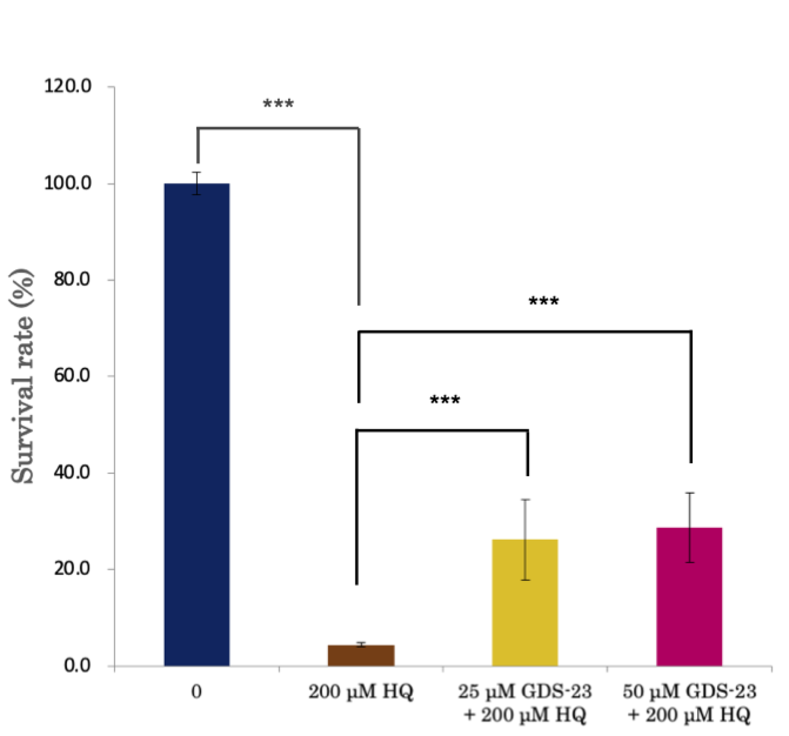

Supplement: S5 Fig — NHEKs were pretreated either with vehicle or GDS-23 (25 or 50 μM) for 24 h and subsequently treated with 200 μM hydroquinone. Cytotoxicity was measured using the neutral red assay and expressed as a percentage relative to the control cells. Results are expressed as mean ± S.D. (n = 6). Significance; ***p < 0.001 (Tukey’s test). GDS-23, glyceryl polyethylene glycol-23 distearate; NHEK, normal human epidermal keratinocyte. (TIF) [file pone.0291905.s005.tif]
