# Supplementary material for: Noncanonical mechanism of Nrf2 activation by diacylglycerol polyethylene glycol adducts in normal human epidermal keratinocytes
Source: PLoS One. 2023 Oct 11;18(10):e0291905. doi: 10.1371/journal.pone.0291905 (PMC10566712; doi:10.1371/journal.pone.0291905)
Supplement: Attachment — Submitted filename: Response to Reviewers ver.2.docx [file pone.0291905.s007.docx]

Response to Reviewers

Dear Editor

I am writing to inform you of the corrections made to the reference list of our manuscript in accordance with the journal's requirements.

1. We identified an error in the patent number of one of our references and have made the necessary correction.

2. Regarding reference number 2, the absence of a DOI number made it unclear whether the paper had been retracted. To avoid any ambiguity, we have replaced it with another paper from the same author group.

3. Additionally, we noticed that the display format of the DOI numbers in our references was not consistent. We have now standardized it for clarity.

Please review the updated reference list and let us know if there are any further issues. We appreciate your guidance and patience throughout this process.

Dear Reviewer #2

Thank you for your constructive feedback on our manuscript. In response to Reviewer #2's suggestions, we have made the following revisions: As recommended, we have added a section in the discussion addressing the potential of using 3D tissue culture as a future direction for our study. (page 24, lines 416 - 417) We hope these revisions address the reviewer's concerns adequately.

Best regards,

Tatsuro Miyoshi
